# The prognostic importance of a history of hypertension in patients with symptomatic heart failure is substantially worsened by a short mitral inflow deceleration time

**DOI:** 10.1186/1471-2261-12-30

**Published:** 2012-04-25

**Authors:** Charlotte Andersson, Gunnar H Gislason, Peter Weeke, Jesper Kjaergaard, Christian Hassager, Dilek Akkan, Jacob E Møller, Lars Køber, Christian Torp-Pedersen

**Affiliations:** 1 Department of Cardiology, Gentofte Hospital, Hellerup, Denmark; 2 The Heart Centre, Rigshospitalet, Copenhagen, Denmark; 3 Department of Cardiology, Copenhagen University Hospital, Gentofte Niels Andersens Vej 65, DK 2900, Hellerup, Denmark

## Abstract

**Background:**

Hypertension is a common comorbidity in patients with heart failure and may contribute to development and course of disease, but the importance of a history of hypertension in patients with prevalent heart failure remains uncertain.

**Methods:**

3078 consecutively hospitalized heart failure patients (NYHA classes II-IV) were screened for the EchoCardiography and Heart Outcome Study (ECHOS). The left ventricular ejection fraction (LVEF) was estimated by 2 dimensional transthoracic echocardiography in all patients and a subgroup of 878 patients had additional data on pulsed wave Doppler assessment of transmitral flow available. A restrictive filling (RF) was defined as a mitral inflow deceleration time ≤140 ms. Patients were followed for a median of 6.8 (Inter Quartile Range 6.6-7.0) years and multivariable Cox regression models were used to assess the risk of all-cause mortality associated with hypertension.

**Results:**

The study population had a mean age of 73 ± 11 years. 39% were female, 27% had a history of hypertension and 48% had a RF. Over the study period, 64% of the population died. Hypertension was not associated with increased risk of mortality, hazard ratio (HR) 0.95 (0.85-1.05). LVEF did not modify this relationship (p for interaction = 0.7), but RF pattern substantially influenced the outcomes associated with hypertension (p for interaction < 0.001); HR 0.75 (0.57-0.99) and 1.41 (1.08-1.84) in patients without and with RF, respectively.

**Conclusions:**

In patients with symptomatic heart failure, a history of hypertension is associated with a substantially increased relative risk of mortality among patients with a restrictive transmitral filling pattern.

## Background

Hypertension is a common comorbidity in patients with heart failure and may contribute to development and course of disease, but the importance of a history of hypertension in patients with prevalent heart failure remains uncertain. Several studies have found a history of hypertension to be without any prognostic importance [1-3] or even to be associated with a decreased risk of mortality [4]. In order to better understand this relationship, we investigated the outcomes associated with a history of hypertension in an unselected cohort of patients hospitalized with symptomatic heart failure with emphasis on the prognosis as dependent on left ventricle systolic and diastolic function.

## Methods

The EchoCardiography and Heart Outcome Study (ECHOS) was a multicenter double-blind clinical trial performed in Denmark, Norway and Sweden with the purpose of comparing placebo to nolomirole 5 mg (a pre-synaptic stimulator of DA_2_-dopaminergic and alpha _2_-adrenergic receptors in peripheral sympathetic nerve endings) in patients with heart failure [5]. Nolomirole was found to have no impact on mortality and for the present analyses, we included all patients screened for entrance in ECHOS [5]. To be eligible for screening, patients were required to receive treatment with diuretics, to be in New York Heart Association (NYHA) class II-IV and to have had symptoms corresponding to NYHA classes III-IV during the preceding month. Patients with acute pulmonary edema, uncorrected hemodynamically significant obstructive valve disease, clinically significant obstructive cardiomyopathy, or acute myocardial infarction (AMI) or cardiac revascularization within the preceding month were excluded.

During the screening process, a full clinical examination and a transthoracic echocardiography were obtained for all patients. The echocardiography investigation did as a minimum include two dimensional records from the parasternal (long and short axis) and apical views (two chamber, four chamber and apical long-axis) in order to evaluate the systolic function. In addition, investigators were encouraged to obtain measurements of mitral inflow (by pulsed wave Doppler in the apical four-chamber view). All echocardiogram records were sent to a core laboratory for evaluation. The systolic function was estimated through the use of wall motion index (WMI) and the left ventricular ejection fraction (LVEF) was subsequently calculated using a 16 segment reverse scoring system, as previously described [6]. Measurements of transmitral filling pattern (i.e., peak flow velocity and deceleration time in early diastole [E wave] and peak flow velocity in atrial contraction [A wave]) were obtained as averages of 5 consecutive cardiac cycles for patients in sinus rhythm and 10 cardiac cycles for patients in atrial fibrillation. A restrictive left ventricular filling pattern was considered present if transmitral deceleration time was below or equal to 140 ms [7]. Atrial fibrillation was no exclusion criteria, because a short deceleration time has shown to be of similar prognostic importance in patients with and without atrial fibrillation [8-10]. A history of hypertension was defined by medical history and required current or previous antihypertensive treatment. Patients were considered to have hyperlipidaemia if they were medically treated or had a total cholesterol > 5 mmol/l or LDL > 3 mmol/l. Creatinine clearance was estimated using the Modification of Diet in Renal Disease (MDRD) study equation [11].

Patients were screened in the period of 2001–2002 and vital status (all-cause mortality) was obtained from the Danish National Person Registry in November 2008.

### Ethics

The study was performed in conformity with the Declaration of Helsinki III and was approved by the Danish central ethical committee. All patients gave their written, informed consent before participating.

### Statistics

Continuous variables were compared with Kruskal-Wallis test and discrete variables with chi-square test. Mortality curves were generated using the Kaplan-Meier estimators and test of equality over strata was performed using the log-rank test. Cox proportional hazard models were used for analyses of the relative risk of all-cause mortality. All models included the following covariates: gender, age, smoking, and a history of the following conditions: hyperlipideamia, ischemic heart disease, stroke, diabetes, chronic obstructive pulmonary disease, and atrial fibrillation. The prognostic importance of a history of hypertension as dependent on LVEF, a restrictive filling pattern and left ventricle posterior wall diameter, respectively, was tested by inclusion of variables plus an interaction term in overall Cox analysis and subsequently analyzed in stratified subgroups.

## Results

In total 3078 patients were screened. Of these 878 patients had complete measurements of the mitral inflow, LVEF and hypertension status. (880 patients had measurements of mitral inflow, but two of these lacked data on hypertension status). Baseline characteristics for overall population and for patients with and without hypertension are shown in Table 1, and for patients with and without hypertension stratified by mitral filling pattern in Table 2. Overall, the mean age was 75 (±standard deviation 11) years, 39% were female and 27% had hypertension. Of the subgroup with data on deceleration time, 135 patients with hypertension and 318 patients without hypertension had a non-restrictive filling pattern, whereas 122 and 303 patients with and without hypertension had a restrictive filling pattern. The four groups resembled each other in most variables, although there were small differences in the prevalence of diabetes mellitus, LVEF and pharmacological treatment between the groups.

**Table 1:** Baseline characteristics

	***Overall population***	***Hypertension***	***No hypertension***
	***N = 3078***	***N = 817 (27%)***	***N = 2255 (73%)***
Age (years)	75 (53;89)	75 (54;89)	75 (53;89)
Male gender*	61%	57%	62%
BMI (kg/m^2^) *	25.8 (18.7;36.2)	26.8 (19.3;38.0)	25.3 (18.5;35.6)
Creatinine clearance (mL/min/1.73 m^2^)	58 (25;97)	57 (23;99)	58 (27;96)
Wall motion index *	1.3 (0.5;2.0)	1.5 (0.5;2.0)	1.3 (0.5;2.0)
LVEF ≤45% *	60%	55%	62%
Smokers	29%	29%	30%
Diabetes mellitus *	15%	21%	13%
Hyperlipidaemia *	32%	36%	30%
History of ischemic heart disease	44%	42%	44%
Previous stroke or TIA *	11%	15%	10%
Atrial fibrillation	35%	35%	35%
COPD	24%	23%	24%
*Medications at discharge:*			
Betablockers *	43%	51%	41%
ACE inhibitors	56%	57%	55%
Angiotensin II blockers *	7%	12%	5%
Calcium channel blockers *	14%	19%	12%
Diuretics	93%	92%	93%
Statins	18%	20%	18%
Nitrates	20%	19%	20%
Glycosides (digoxin)*	38%	34%	39%
Oral anticoagulants*	30%	27%	32%

Footnote: Continuous variables are presented as median 5^th^; 95^th^ percentiles.* indicates p for difference between the two sub-groups <0.05.

**Table 2:** Baseline characteristics of patients with measurements of mitral inflow available

	***Hypertension (n = 257)***		***No hypertension (n = 621)***	
	**Restrictive (n = 122)**	**Non-restrictive (n = 135)**	**Restrictive (n = 303)**	**Non-restrictive (n = 318)**
Age (years)	74 (52;87)	75 (55;90)	74 (52;90)	76 (53;90)
Male gender	60%	55%	61%	61%
BMI (kg/m^2^) *	27.2 (18.0;35.3)	26.5 (19.5;36.0)	25.3 (18.4;35.3)	25.0 (18.4;36.7)
Decelleration time (ms) *	117 (87;138)	187 (145;278)	116 (84;137)	171 (143;296)
E/A ratio *	1.8 (0.6;3.4)	1.0 (0.6;2.6)	1.5 (0.6;3.1)	1.0 (0.6;3.1)
Creatinine clearance (mL/min/1.73 m^2^)	58 (18;97)	57 (21;99)	63 (29;100)	57 (26;96)
Wall motion index *	1.3 (0.5;2.0)	1.6 (0.6;2.0)	1.1 (0.5;2.0)	1.4 (0.6;2.0)
LVEF ≤45% *	64%	48%	75%	58%
Left ventricle posterior wall thickness (mm) *	12 (9;17)	11 (8;15)	11 (7;15)	10 (7;14)
Smokers	33%	28%	33%	24%
Diabetes mellitus *	19%	22%	13%	12%
Hyperlipidaemia	37%	30%	30%	30%
History of ischemic heart disease	44%	45%	44%	44%
Previous stroke or TIA	15%	17%	11%	10%
Atrial fibrillation	44%	35%	38%	38%
COPD	19%	29%	20%	24%
*Medications at discharge:*				
Betablockers *	51%	59%	43%	47%
ACE inhibitors	55%	59%	65%	56%
Angiotensin II blockers *	12%	10%	5%	6%
Calcium channel blockers *	15%	25%	11%	12%
Diuretics	92%	96%	93%	92%
Statins	20%	21%	17%	20%
Nitrates *	11%	25%	16%	20%
Glycosides (digoxin)*	42%	27%	42%	37%
Oral anticoagulants	28%	24%	36%	31%

Footnote: Continuous variables are presented as median 5^th^; 95^th^ percentiles.* indicates p for difference between the four sub-groups <0.05.

### Survival analyses

**Figure 1:**
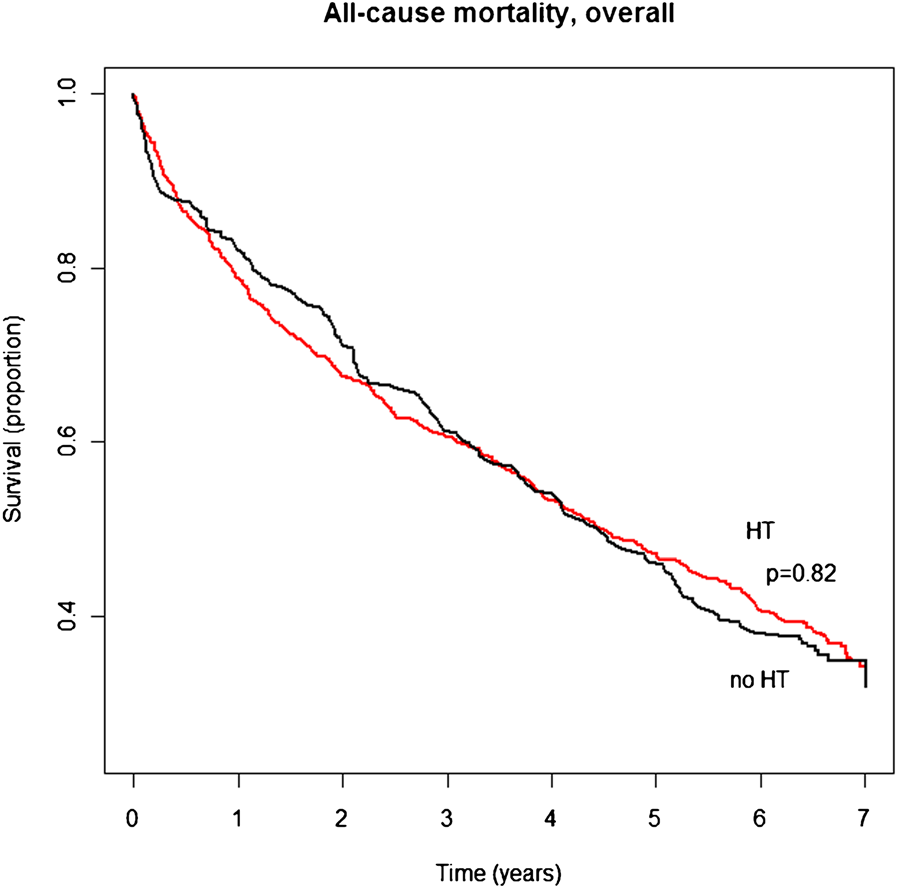
Unadjusted mortality rates for patients with and without hypertension (HT).

Over a median follow-up time of 6.8 (Inter Quartile Range 6.6-7.0) years, 67% of the population died. Unadjusted mortality rates for patients with and without hypertension are shown in Figure 1. In overall multivariable Cox analysis, hypertension was associated with a hazard ratio (HR) of 0.95 (95% Confidence Interval [CI] 0.85-1.05) and was not found to be modified by LVEF (p for interaction between LVEF and hypertension = 0.7). In separate analyses of patients with and without a restrictive filling pattern, the LVEF was without importance for the prognosis associated with hypertension in both subgroups (p for interactions = 0.3 and 0.7 in subgroup with and without restrictive filling pattern, respectively).

**Figure 2:**
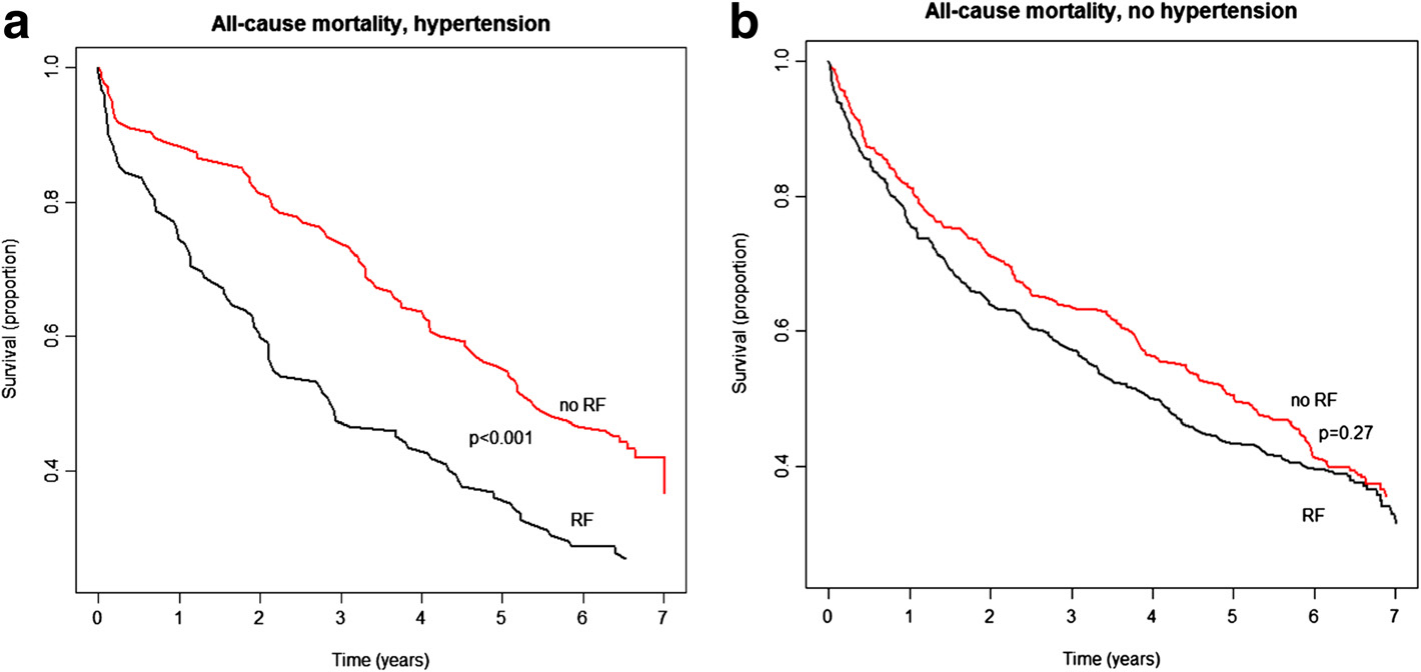
**a: Unadjusted mortality rates in patients with hypertension, stratified by a restrictive filling (RF). b: **Unadjusted mortality rates in patients without hypertension, stratified by a restrictive filling (RF).

**Figure 3:**
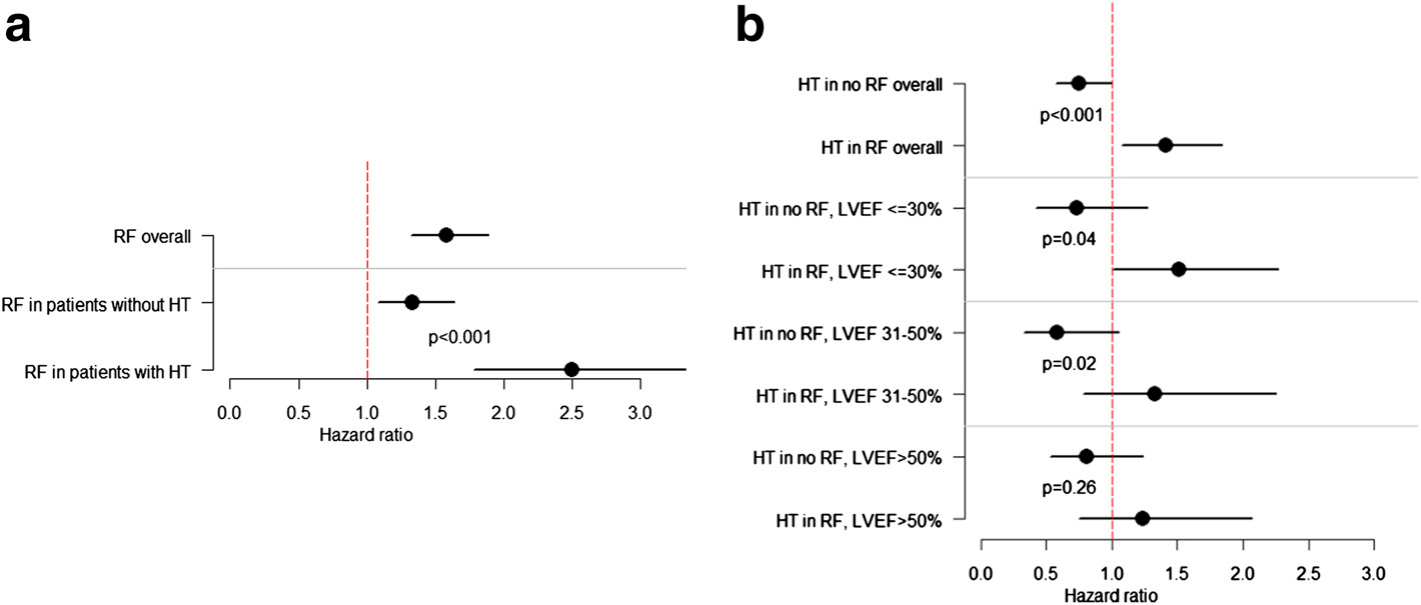
**a: Hazard ratios associated with a restrictive filling pattern (RF) overall and in patients with and without a history of hypertension (HT).b: **Hazard ratios associated with hypertension (HT) in different subgroups of patients. RF = restrictive filling pattern; LVEF = left ventricular ejection fraction.

Figure 2 a + b presents Kaplan-Meier curves for patients with and without a restrictive filling pattern stratified by hypertension status. As previously shown [7], having a restrictive filling pattern was associated with an increased risk of mortality, but importantly this relative risk was substantially higher among patients with hypertension than among patients without hypertension (Figure 3a). Further, the transmitral filling pattern was shown to substantially influence the outcomes associated with hypertension; p for interaction <0.001; Figure 3b. A history of hypertension was associated with 25% decrease in relative risk of mortality (HR 0.75 [0.57-0.99]) among those with a non-restrictive filling pattern and a 41% increase in relative risk of mortality (HR 1.41 [1.08-1.84]) among those with a restrictive filling pattern. As Figure 3b also illustrates, the hazard ratios associated with hypertension in relation to the mitral filling pattern were not dependent on LVEF. In subgroup analysis of patients with available measurements on left ventricle posterior wall diameter (n = 753), no interaction was found between hypertension and left ventricle posterior wall (p = 0.4).

## Discussion

The present study demonstrated that the prognostic importance of a history of hypertension in unselected heart failure patients is dependent on the diastolic, but not the systolic, function. We found that in patients with a restrictive filling pattern (i.e., a mitral deceleration time <140 ms), a history of hypertension was associated a substantial increase in relative risk of mortality (HR = 1.41 [1.08-1.84]). This relationship was established across the whole LVEF spectrum and LVEF did not modify the prognosis associated with a history of hypertension. For comparison, such an increase is of similar magnitude as e.g. the risk carried by diabetes [12]. Thus, the presence of hypertension in patients with heart failure and restrictive transmitral filling pattern, irrespectively of LVEF, merits increased attention and these patients may be candidates for more aggressive anti-hypertensive pharmacotherapy in order to improve prognosis. However, the benefit of anti-hypertensive treatment in this group of patients remains to be established and e.g. the Perindopril in Elderly People with Chronic Heart Failure (PEP-CHF) study, which compared perindopril to placebo in patients with preserved LVEF and diastolic dysfunction (mean deceleration time approximately 200 ms), found no effect on mortality [13].

An important limitation of the present study was that we had no data on actual blood pressure values and it is possible that patients included in our study were adequately regulated in blood pressure. Thus, the mechanisms underlying our findings of an adverse prognosis associated with a history of hypertension only in patients with restrictive filling may also have related to other factors than poor anti-hypertensive treatment. In this context, hypertension may lead to left ventricular hypertrophy and remodeling in some patients, but not in others, and a differential response to anti-hypertensive treatment on left ventricle remodeling has been demonstrated in one previous study [14]. In this latter study, those hypertensive patients who responded to anti-hypertensive pharmacological treatment with a reduction in left ventricular mass were shown to have a more favorable prognosis than patients who did not respond with a reduction in left ventricular mass [14].

Another possible mechanism explaining our findings may relate to severe systemic hypertension leading to pulmonary hypertension through increased end-diastolic pressure, and the presence of pulmonary hypertension in patients with heart failure has shown to be a strong and independent adverse prognosticator [15]. In this context, it is well-known that high right ventricular pressure may interact with left ventricular pressure, leading to a short deceleration time [16]. Further supporting this theory, a short deceleration time has shown to have a close correlation with pulmonary capillary wedge pressure in patients with left ventricular systolic dysfunction [17].

Finally worth discussing, in the subgroup of patients without a restrictive filling, we found a paradoxical low relative risk of mortality associated with a history of hypertension (HR = 0.75 [0.57-0.99]). It is indeed well-known that high blood pressure at time of heart failure admission is associated with a paradoxical protective effect on the risk of mortality [18-21]. Possibly, such a paradoxical effect may be driven by more severe cardiac dysfunction leading to a decline in systemic blood pressure, thereby making low blood pressure a marker of more advanced heart failure. A similar protective effect on mortality risk in heart failure cohorts is known for other classical risk factors for cardiovascular morbidity and mortality, e.g. obesity and hypercholesterolaemia and the reasons have been intensively discussed [18].

### Limitations

Some important limitations should be noted. As previously mentioned, data on exact blood pressure values were not available for the present study and the diagnosis of hypertension relied on patient history and anti-hypertensive treatment. LVEF was estimated by WMI, which to some extent is observer-dependent and only an approximation of LVEF. It cannot be excluded that the lack of interaction between left ventricle posterior wall diameter and a history of hypertension may be due to insufficient power, because measurements of left ventricle posterior wall diameter were available in approximately one third of all patients. Furthermore, measurements of transmitral flow were not mandatory for the ECHOS trial and only a subgroup of 880 patients had measurements available. These patients resembled the other patients, but it cannot fully be excluded that this subgroup differed in unmeasured characteristics from the rest of the ECHOS screening population. Unfortunately, we did not have measurements from tissue Doppler or other modern echocardiographic modalities available for analyses (they do however not as yet allow analyses of long-term outcomes). Finally, we did not have causes of deaths available for the present analyses, but from others’ work we know that the majority of patients with heart failure (NYHA II-IV) die from pump failure or arrhythmias [22].

## Conclusion and clinical implication

In patients with symptomatic heart failure, a history of hypertension is associated with a substantially increased relative risk of mortality among patients with a restrictive transmitral filling pattern. An increased awareness of this poor prognosis is warranted and these patients may be candidates to more aggressive anti-hypertensive pharmacotherapy in order to improve prognosis. However, the effect of anti-hypertensive treatment in this patient-group is as yet not well-investigated.

## Competing interests

The authors declare that they have no competing interests.

## Authors’ contributions

CA, LK, JK, CH, and CTP contributed with the study design. CA made the statistical analyses and wrote the initial draft. All authors (CA, GG, PW, JK, CH, DA, JEM, LK, and CTP) revised it critically for important intellectual content. All authors read and approved the final manuscript.

## Pre-publication history

The pre-publication history for this paper can be accessed here:

http://www.biomedcentral.com/1471-2261/12/30/prepub
